# Effect of Internet peer-support groups on psychosocial adjustment to cancer: a randomised study

**DOI:** 10.1038/sj.bjc.6605646

**Published:** 2010-04-27

**Authors:** M T Høybye, S O Dalton, I Deltour, P E Bidstrup, K Frederiksen, C Johansen

**Affiliations:** 1 Department of Psychosocial Cancer Research, Institute of Cancer Epidemiology, Danish Cancer Society, Copenhagen, Denmark; 2 Department of Statistics and Epidemiology, Institute of Cancer Epidemiology, Danish Cancer Society, Copenhagen, Denmark

**Keywords:** randomised controlled trial, Internet support, survivorship, rehabilitation, psychological measures

## Abstract

**Background:**

We conducted a randomised study to investigate whether providing a self-guided Internet support group to cancer patients affected mood disturbance and adjustment to cancer.

**Methods:**

Baseline and 1-, 6- and 12-month assessments were conducted from 2004 to 2006 at a national rehabilitation centre in Denmark. A total of 58 rehabilitation course weeks including 921 survivors of various cancers were randomly assigned to a control or an intervention group by cluster randomisation. The intervention was a lecture on the use of the Internet for support and information followed by participation in an Internet support group. Outcome measures included self-reported mood disturbance, adjustment to cancer and self-rated health. Differences in scores were compared between the control group and the intervention group.

**Results:**

The effect of the intervention on mood disturbance and adjustment to cancer showed a transient difference at the 6-month follow-up, where the intervention group reported less reduction in anxious preoccupation (*P*=0.04), helplessness (*P*=0.002), confusion (*P*=0.001) and depression (*P*=0.04). Otherwise no significant effects were observed.

**Conclusion:**

We conclude that use of Internet-based support groups in cancer patients still needs to confirm long-lasting psychological effects.

During the past decade, the Internet has become a widely used resource for information on cancer and for support (Eysenbach *et al*, 2004). Small randomised studies suggest that introducing Internet-based support to cancer survivors may result in significant positive outcomes regarding social support, competence in finding information (Gustafson *et al*, 2001), depression (Winzelberg *et al*, 2003) and self-perceived health status (Owen *et al*, 2005) (Online Supplementary Table 1). The overall quality of life of cancer patients has not been shown to be improved (Gustafson *et al*, 2001; Winzelberg *et al*, 2003; Owen *et al*, 2005); however, explorative studies describe that Internet groups empower cancer patients and facilitate new social networks (Lieberman *et al*, 2003; Høybye *et al*, 2005). Internet interventions in previous randomised studies all provided a discussion forum (Gustafson *et al*, 2001; Winzelberg *et al*, 2003; Owen *et al*, 2005) and further included various information services (Gustafson *et al*, 2001; Owen *et al*, 2005), cancer decision services (Gustafson *et al*, 2001), structured coping skills training exercises (Owen *et al*, 2005) and the keeping and sharing of personal journals (Gustafson *et al*, 2001; Winzelberg *et al*, 2003; Owen *et al*, 2005).

Use of the Internet by Danish society is among the highest in the world, with 83% of the population having access in 2004 (Statistics Denmark, 2005) and intensive use for health information (European Opinion Research Group (EORG) and Spadaro, 2003). We hypothesised that a self-guided support group for cancer patients on the Internet following a larger rehabilitation programme would positively affect rehabilitation as measured by a decrease in psychological distress and an increase in adjustment to cancer and self-rated health.

## Materials and Methods

### Participants

Cancer survivors participating in a public rehabilitation programme at the national Rehabilitation Centre Dallund in Denmark were eligible for the study, except for participants in courses where survivors more than 50 years of age were targeted specifically.

### Study design

This two-arm randomised trial assessed the incremental effect of participation in an Internet-based peer-support group following a week-long rehabilitation programme, over and above any possible general effect of participation in the rehabilitation programme. Following baseline assessment of socio-demographic characteristics, use of the Internet and psychological measures, all individuals organised in groups of ⩽20 persons participated in a rehabilitation programme comprising a 6-day retreat. The retreat offers a combination of lectures and patient group work on themes related to survivorship concerns of psychological, existential and physical late effects. The programme is conducted by a multi-disciplinary professional team. Course weeks are commonly organised around a shared issue in relation to survivorship, like ‘returning to work’ or around a particular cancer diagnosis or age group, so that the group of cancer survivors in a given week will share some common concerns. Cancer survivors from all regions in Denmark participate at their own or their doctor's initiative and undergo no formal screening before participation in the rehabilitation programme (Høybye *et al*, 2008).

All individuals in a rehabilitation course allocated for intervention attended a 2 h introductory lecture at the rehabilitation centre given by a member of the research team (MTH). The lecture gave instructions on how to use the Internet for information on cancer, and participants were invited to participate in an Internet peer-support group.

The control weeks received no treatment beyond the ordinary rehabilitation programme and the 2 h programme slot in control course weeks was recreational time with no specific programme offered.

At baseline, written consent was obtained from all participants. Data management and security regarding this study were approved by the Danish Data Protection Agency.

Participants in groups assigned to both conditions were followed up individually at 1, 6 and 12 months by self-reported assessments of quality of life indicators.

### Randomisation

To ensure that all participants in the same course were allocated to the same condition, we carried out the randomisation by clusters of course weeks and not by individual. The centre planned themes and focus areas of the course weeks 6 months in advance, providing us the possibility only to randomise courses each half year and not the total number of courses at once. On the basis of the course schedules provided by the staff at the rehabilitation centre, we randomised 58 course weeks at the centre.

**Figure 1:**
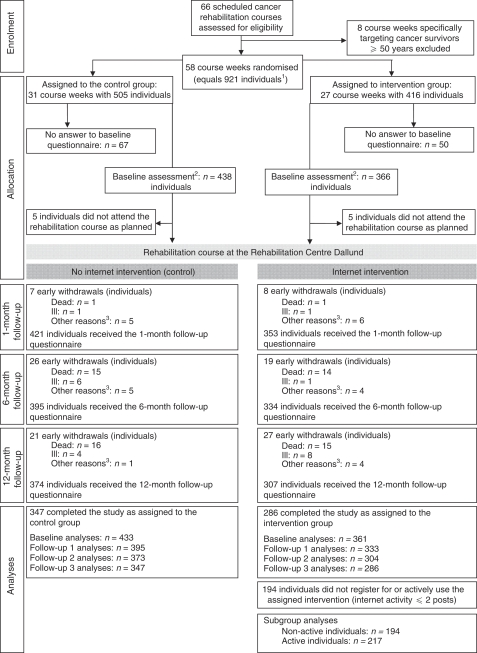
Randomisation and follow-up of cancer survivors eligible for participation in the study of Internet support in cancer rehabilitation, Denmark, 2004–2006. All follow-up questionnaires were mailed to all participants who completed baseline and who were alive and had not withdrawn from study. ^1^All participants eligible to participate in this study attended a week-long rehabilitation course at the Dallund Rehabilitation Centre, Denmark, between 19 April 2004 and 31 December 2005. Cancer survivors from all regions in Denmark participated at their own or their doctor's initiative and underwent no formal screening before participation. ^2^Baseline assessment was carried out 2 weeks before attending the rehabilitation course. ^3^Includes illness in family, lack of energy to participate or emigration.

Assignment of course weeks to either intervention or control group was carried out as a sealed-envelope procedure organised by a computer program as 27 intervention weeks and 31 control weeks (Figure 1). Following the randomisation procedure the assignment was disclosed to the research team, as the team was also in charge of teaching the lecture on Internet use to the intervention group that could therefore not be masked. Individuals became aware of their group assignment once they received the program for their specific course week.

### Intervention Internet peer-support groups

The Internet peer-support groups were intended as a space for maintaining the relations that were established during the week-long rehabilitation course. The groups provided a self-guided space for communication, including an Internet discussion forum, a live chat room and a personal message system. No therapeutic content or information services were offered within the groups.

All Internet groups were run in a browser-based software platform with access through the website of the Danish Cancer Society. All discussions were encrypted and protected by a password. The Internet groups were all closed groups, accessible only by an invitation received by e-mail after registration for the study and were open to access 24 h a day for 13 months from their individual starting date set to the date of the Internet lecture provided at the rehabilitation centre in all groups. Depending on the topic for each week at the rehabilitation centre, Internet groups would form around a shared cancer diagnose or particular shared concern in relation to the experience of cancer.

Activity levels in the Internet support groups, measured as the number of postings in the Internet groups, were counted and collected monthly during the study. The web-based system did not include a statistical application to track the use of the groups, log-on, and so on, which meant that the number of posts would be counted manually in each group. Active use of the Internet groups was in this study defined as having accepted the invitation for the group, created a user profile and posting ⩾2 messages to a group. Owing to the lack of a statistical module in the web-based group system, activity beyond posting cannot be accounted for in our analysis.

### Study end points

The primary end points were differences in changes in psychological distress and adjustment to cancer. Secondary end points were differences in changes in self-perceived health. Table 1 describes each measure.

### Statistical analysis

On the basis of experience from similar analyses, we expected that a sample size of 1000 persons would be adequate and realistic in terms of power within the timeframe of the study. As we lacked information on the magnitude of the anticipated effect, no formal power calculations were performed.

Statistical analyses were conducted on an intention-to-treat basis using Stata 9 (Stata Statistical Software, 2005, College Station, TX, USA) with the option cluster to adjust the standard errors for intra-group correlation. Analyses were conducted by group assignment to intervention or control, based on individual reports within each group.

Primary analyses on baseline characteristics of the groups were compared by *t*-tests or by Fisher's exact tests if any of the expected cell counts was less than five. Further, primary analyses used linear models adjusting for sex, age, diagnosis group, education, marital and employment status, and for clustering of subjects within weeks of the rehabilitation course, hence allowing subjects who attended the same course to be more similar among themselves than to subjects from different groups. To evaluate the impact of the lecture on Internet use and the availability of Internet peer-support groups, we computed amount of change for the entire population and intervention and control conditions on levels of change. However, we did not impose any strict hierarchical structure on the data. Differences between scores on baseline and follow-up measures were the assessment of effect considered, so that positive values indicated increased scores. As we did not have *a priori* hypotheses on the timing of the effect, the three follow-up times were analysed separately. The same tests as in the comparison of the baseline scores were used with additional adjustment on the baseline score itself.

To evaluate if intervention outcomes were mediated by socio-demographic differences, we stratified analyses by gender, age groups, marital status, education level and baseline score using interaction tests. As to the baseline score of each measure, the 10% of the individuals doing worst at baseline was identified, and the impact of the intervention for these was compared with the impact of the intervention for the remaining 90%. These analyses were also adjusted for baseline score as a linear variable.

Secondary analyses using linear models as described above compared individuals in the intervention group who actively used the Internet support groups (wrote ⩾2 posts) with non-active participants in the intervention group and with controls to evaluate the impact of the support groups. Further, secondary analyses aimed at describing the effect of the activity of posting in Internet peer-support groups on the subgroup of active participants. Linear regression models were used to evaluate the impact of the total number of postings exchanged within the group, the activity per person and the number of persons in the Internet group on the outcome measures.

## Results

### Study population

A total of 66 scheduled rehabilitation course weeks between 19 April 2004 and 31 December 2005 were screened for eligibility (Figure 1). All 58 eligible courses including 921 individuals were enrolled and randomised as 27 intervention weeks (*n*=416 persons) and 31 control weeks (*n*=505 persons). Baseline assessments were obtained from 799 individuals. Data from 10 individuals (5 from each treatment group) were subsequently excluded, as they did not attend a rehabilitation course week as planned, leaving a final study population of 794 individuals analysed by ‘intention-to-treat’ basis (intervention: *n*=361 out of 794; control: *n*=433 out of 794). Participants were followed up for 12 months in accordance with their dates of attendance of the rehabilitation course.

The distribution of the demographic characteristics in the two conditions at baseline was significantly different, the groups assigned to intervention were containing more men, younger persons and more cohabiting persons (Table 2). Generally, the participants were well educated, working and used the Internet at baseline. No baseline difference was found on previous use of Internet support (Table 2).

### Attrition and intervention adherence

As shown in Figure 1, participants in the intervention and control groups showed similar attrition rates during the 12-month study (15% of control participants and 22% of intervention participants). All 27 intervention course weeks (*n*=361 individuals) participated in the lecture on Internet use at the rehabilitation centre. Of the 27 intervention course weeks, participants from 26 courses decided to start an Internet-based peer-support group, with 60% (*n*=217 out of 361) of participants accepting the invitation to join a support group and posting at least two messages on the online group system. The Internet groups comprised 2–18 cancer survivors. The total number of messages posted (*n*=2154) ranged from 2 to 241 in the Internet groups. Ten groups included 2–6 participants, and over the first 2 months, these exchanged 2.4 posts per participant on average. The 16 larger groups, including more than seven participants, exchanged 3.5 posts per participant over the same period. Posting activity peaked in the first 3 months and then decreased.

Compared with users, non-users of the Internet support groups were more likely to be men (22 *vs* 11% *P*=0.005), older (40 *vs* 10% >60 years; *P*=<0.001), single (38 *vs* 22% *P*=0.002), lower educated (22 *vs* 7% only basic education; *P*=<0.001), without active affiliation with the working market (48 *vs* 78% employed; *P*=<0.001) and not using the Internet at baseline (59 *vs* 16% *P*=<0.001).

### Primary outcomes

Table 3 shows the adjusted differences change in scores compared to baseline level for each study outcome at each follow-up point.

The effect of the intervention on coping and adjustment to cancer showed a transient difference at the 6-month follow-up, when the intervention group reported more anxious preoccupation (*P*=0.04) and helplessness (*P*=0.002) (Table 3). We found no effect of the intervention on total mood disturbance or on any of the POMS subscales at any time, except for a transient difference on the subscales confusion/bewilderment (*P*=0.001) and depression/dejection (*P*=0.04) at the 6-month follow-up, indicating more confusion and less improvement in depression in the intervention group than in the control group (Table 3). However, the intervention group reported a significantly higher increase on vigour/activity (*P*=0.001) at 12-month follow-up.

### Secondary outcomes

Self-rated global health in the intervention group was not significantly different to the control group at any of the follow-up times (Table 3).

### Potential confounders

To determine whether intervention effects varied as a function of socio-demographic characteristics or baseline score, we analysed the possible interactions. Results did not show any differential impact of the intervention on any primary or secondary outcomes as a function of sex, marital status, employment or education. We did not have access to logging the number of visits to the groups, and were therefore not able to elucidate the function of passive user behaviour.

### Subgroup analyses

The 217 participants in the intervention group who actively used an Internet support group reported a significant increase in fighting spirit at 1- (*P*=0.03), 6- (*P*=0.05) and 12-month (*P*=0.04) follow-up whereas the non-active participants (*n*=194) reported a decrease at 6- and 12-month follow-up (Table 4). Active participants did, however, report significantly poorer self-rated global health at baseline (*P*=0.03; Table 4).

### Adverse effects

No study participants reported adverse events. However, data from an associated online focus-groups study (conducted by MTH and PEB) drew attention to possible events of anxiety in the active users of the Internet support groups. On the basis of this, we analysed the changes in mood disturbance in relation to number of messages posted. These analyses showed that an increased number of postings in an Internet group were associated with slightly changed mood disturbance, but significant only at 12 months (*β* coefficient per 10 postings, 0.57; 95% CI, 0.28–0.86). The number of postings was significantly associated with an increased level of depression/dejection (*β* coefficient per 10 postings, 0.13; 95% CI, 0.07–0.19), fatigue/inertia (*β* coefficient per 10 postings, 0.13; 95% CI, 0.03–0.22) and tension/anxiety (*β* coefficient per 10 postings, 0.11; 95% CI, 0.06–0.16) as measured by POMS at 12-month follow-up (Online Supplementary Table 2).

## Discussion

Results from this randomised study did not show a positive effect of participation in an Internet-based peer-support group following a week-long rehabilitation programme on mood disturbance, adjustment to cancer or self-rated global health status. In general, psychological well-being improved over time in both the intervention and the control groups. This may reflect an effect of the week-long rehabilitation programme in which both groups participated. Limited, transient differences between the two groups were seen, but these were mainly due to less improvement in psychological well-being in the intervention group. Secondary analyses showing more fighting spirit in participants in the intervention group who used the Internet support group than in participants not using the groups could however suggest that fighting spirit is an element of rehabilitation, which may be strengthened by the social interaction in a peer group.

In line with our study results, Owen *et al* (2005) reported no significant, major effects of a self-guided Internet coping group for women with breast cancer. It is possible that some effect of Internet-based cancer support groups depend on active, professional moderation, as mentioned in previous studies (Gustafson *et al*, 2001; Winzelberg *et al*, 2003). However, the experimental conditions in studies like this do not possibly provide a social environment for supportive interaction that is comparable to that of self-generating cancer support groups on the Internet, which relies of a complex social process (Lieberman, 2004; Lieberman and Goldstein, 2005). Results from this type of study may therefore not apply to self-generated support groups on the Internet.

Women with high emotional support from partners have previously shown adverse effects of a peer-support intervention, suggesting a possible association of social resources with effects of the peer-support groups (Helgeson *et al*, 2000). As more participants in the intervention condition in this study were married, this could be another underlying reason for the transient negative effect of the intervention, but we do not have data to elucidate this.

Participants in the intervention group became acquainted with other members of their Internet group during the rehabilitation course, which may have had both adverse and positive effects on the interaction in the Internet groups. On the basis of our study data it is not possible to determine the nature of such effect.

Strengths of this study include the use of a randomised design, a relatively large sample size, the 12-month follow-up and the recruitment of participants from a nationwide public rehabilitation programme open to all cancer patients free of charge.

Limitations of this study include the need to perform cluster randomisation as opposed to randomisation by individual, whereby we ended up assigning a large number of individuals who did not adhere to use of Internet peer-support groups (*n*=194 out of 361) to the intervention condition. These intervention participants were characterised by significantly different socio-demographic status and Internet use at baseline, which confirms previous findings of social inequality in the access to and motivation for the use of Internet peer-support groups in cancer patients (Høybye *et al*, 2010). The recruitment and randomisation of groups further did not allow for attention to or screening for baseline psychological function, which may have had a bearing on the effect of the intervention.

Further, the study is limited by the heterogeneity of cancer diagnoses and treatments and treatment stages, which may have reduced the statistical power. However, this may at the same time improve the generalisability of the findings. Choosing not to include participants in the study based on cancer type or stage of cancer may limit our conclusions, yet we find the mixed study population to provide a more realistic reflection of the population of cancer survivors commonly using Internet groups.

Also, the study is limited by the unequal study groups at baseline regarding sex, age and marital status. We consider the difference to be related to the performance of cluster randomisation but we were able to adjust for these socioeconomic and demographic differences in the multivariate analyses. Further, the interaction analyses did not show the effect of the intervention on our primary end points to be modified by these characteristics.

Finally, generalisability of the findings in this study may be somewhat limited due to the particular design of the intervention extending the interaction and the intervention of a previous rehabilitation programme. Generalisability about the study population is possible to some extent, as patients in this study, compared with previous studies show similar levels of baseline distress (Baker *et al*, 2002). Access to a personal computer and the Internet was needed for participation in the full intervention, which may be a barrier in some groups of cancer survivors (Høybye *et al*, 2010). Thus, future implementation of this intervention should take steps to address the social inequality and Internet access issues.

On the basis of this study and previously published randomised studies that all included fewer than 250 cancer patients, we conclude that use of Internet-based support groups in cancer patients still needs to confirm long-lasting psychological effects.

## Figures and Tables

**Table 1:** Description of measures of primary and secondary end points of the study of Internet support in cancer rehabilitation, Denmark (2004–2006)

**Measure**	**Type of measure**	**Description**
*Primary end points*		
POMS-SF (Shacham, 1983; Curran *et al*, 1995)	Self-report	Measures psychological distress. POMS-SF scale is a 37-item short version of POMS scale (McNair *et al*, 1992) for measuring transient states of six moods or affective states: tension/anxiety, depression/dejection, anger/hostility, vigour/activity, fatigue/inertia and confusion/bewilderment. TMD is assessed as the sum of the scores for these six moods, higher scores representing greater mood disturbance, except for vigour/activity where higher scores indicate lesser mood disturbance and the score of this subscale is subtracted from the sum of the rest to provide the TMD. Studies have shown the scale to be valid and reliable (Baker *et al*, 2002).
Mini-MAC (Watson *et al*, 1994)	Self-report	Measures adjustment to cancer and coping styles as five dimensions of cancer-specific cognitive and behavioural coping: fighting spirit, helplessness or hopelessness, anxious preoccupation, fatalism and cognitive avoidance. The MAC assessment indicates the tendency to cope with the stress of cancer in a particular way (Watson *et al*, 1999) and has previously proved to be a valid, reliable assessment (Grassi *et al*, 2005).
		
*Secondary end points*		
Self-rated global health	Self-report	Assessed from one single-item standard question and categorized as excellent/very good, good or fair/poor. Self-perceived health status has been shown to predict mortality, above and beyond the contribution to prediction made by indices based on the presence of health problems, physical disability and biological or lifestyle risk factors (Idler and Benyamini, 1997).

Abbreviations: Mini-MAC=Mini-Mental Adjustment to Cancer; POMS=short version of the Profile of Mood States; TMD=total mood disturbance.

**Table 2:** Socio-demographic characteristics at baseline of 794 participants in the study of Internet support in rehabilitation, Denmark (2004–2006)

	**Internet intervention**		**Control**		
**Characteristic**	***N***	**%**	***N***	**%**	***P*-value** a
Total	361	100	433	100	
					
*Sex*					
Male	57	16	43	10	0.013
Female	304	84	399	90	
					
*Age (years)*					
<45	70	19	57	13	0.013
45–60	205	57	241	56	
>60	86	24	135	31	
Mean	361	53	433	55	0.004
					
*Marital status*					
Married or cohabiting	252	70	255	59	0.001^b^
Living alone^c^	106	29	176	41	
No data	3	1	2	1	
					
*Education* d					
Basic (ISCED 1–2)	50	14	40	9	NS^b^
Secondary (ISCED 3)	131	36	173	40	
Higher (ISCED 4–6)	178	49	216	50	
Unknown	2	1	2	1	
					
*Employment status*					
Employed	231	64	284	66	NS^b^
Sick leave or unemployed	28	8	28	7	
Retired or other^e^	98	27	116	27	
No data	4	1	5	1	
					
*Type of cancer* f					
Breast	179	50	259	60	NS
Colorectal	24	7	23	5	
Head and neck	18	5	17	4	
Haematological	27	8	25	6	
Female genital tract	42	12	40	9	
Upper gastrointestinal tract	6	2	7	2	
Lung	15	4	13	3	
Prostate	7	2	9	2	
Skin	10	3	8	2	
Urinary cancer	3	1	4	1	
Brain cancer	8	2	3	1	
Other	9	3	8	2	
Unknown	13	4	17	4	
					
*Ever used Internet information during illness*					
Yes	216	60	262	61	NS^b^
No	129	36	153	35	
No data	16	4	18	4	
					
*Generally use Internet*					
Yes	216	60	256	59	NS^b^
No	129	36	158	37	
No data	16	4	18	4	
					
*Use of Internet for information or support on cancer*					
Internet in general	97	27	121	28	NS^b^
by e-mail	44	12	46	11	
by chat, mailing lists, self-help groups	24	7	22	5	
No	178	49	224	52	
No data	18	5	20	5	

Abbreviations: ISCED=International Standard Classification of Education; NS=not significant.

a *χ*^2^-Test or Fisher's exact test.

b Excluding missing data or unknown status.

c Comprises divorced, widowed and single

d Highest educational level achieved, classified according to the ISCED.

e ‘Other’ covers persons not affiliated to a workplace for reasons other than unemployment or illness, e.g. housewife, student, maternity leave.

f Head-and-neck cancer comprises cancers of the mouth, pharynx, larynx and thyroid gland; haematological malignancies comprise leukaemia, lymphoma and myelomatosis; cancer of the female genital organs comprises cancers of the uterus, cervix and ovary; upper gastrointestinal tract cancer comprises cancers of the oesophagus and stomach; urinary tract cancer comprises cancers of the kidney and bladder; other cancers comprise cancers of the testis, liver and male breast, sarcoma, carcinoma and cancers at unspecified sites. Cancer sites registered as reported by participants, except for equivocal answers, for which the first tumour registered in the Danish Cancer Registry was used.

**Table 3:** Baseline level and evolution of mean quality of life indicators in control and intervention groups of 794 participants in the study of Internet support in cancer rehabilitation, Denmark (2004–2006)

	**Control group (*n*=433)**				**Intervention group (*n*=361)**				**Significance level of adjusted tests**			
		**Difference between follow-up and baseline level**				**Difference between follow-up and baseline level**						
**Measure**	**Baseline level**	**1** a	**2** b	**3** c	**Baseline level**	**1** a	**2** b	**3** c	**Baseline**	**1** d	**2** d	**3** d
*Profile of mood states*												
Total mood disturbance	16.8	−4.9	−5.2	−7.7	17.5	−3.6	−3.9	−6.7	NS	NS	NS	NS
Anger/hostility	3.0	−0.7	−0.5	−0.7	3.0	−0.4	−0.3	−0.5	NS	NS	NS	NS
Confusion/bewilderment	4.2	−0.2	−0.6	−0.8	4.2	−0.1	0.1	−0.6	NS	NS	0.001^**^	NS
Depression/dejection	6.3	−1.2	−1.5	−1.9	6.2	−0.7	−0.8	−1.4	NS	NS	0.04	NS
Fatigue/inertia	7.0	−0.8	−0.9	−1.3	7.4	−0.9	−1.0	−1.2	NS	NS	NS	NS
Tension/anxiety	6.2	−0.6	−0.9	−1.3	6.4	−0.8	−0.8	−1.5	NS	NS	NS	NS
Vigour/activity	9.8	1.2	0.9	1.4	9.9	0.6	1.1	1.5	NS	NS	NS	0.001^**^
												
*Mental adjustment to cancer*												
Anxious preoccupation	20.4	−0.9	−1.7	−2.0	20.3	−1.0	−1.0	−1.8	NS	NS	0.04^*^	NS
Avoidance	9.7	0.1	0.1	0.2	9.9	−0.1	0.0	−0.1	0.05	NS	NS	NS
Fatalism	13.3	0.0	−0.3	−0.2	13.4	−0.1	−0.1	−0.3	NS	NS	NS	NS
Fighting spirit	11.5	0.1	0.1	0.2	11.7	0.1	0.0	0.1	NS	NS	NS	NS
Helplessness	13.3	−0.6	−1.0	−0.9	13.1	−0.6	−0.3	−0.9	NS	NS	0.002^*^	NS
Self-rated health	3.3	−0.2	−0.2	−0.3	3.3	−0.1	−0.2	−0.2	NS	NS	NS	NS

Numbers are numbers of persons who completed the baseline questionnaire.

Significant in unadjusted *t*-tests at ^*^P<0.05, ^**^P<0.01, NS, not significant at *P*<0.05. Numbers and NS refer to the *P*-value in the F-test of the difference between groups in a linear regression model adjusted for sex, age, diagnostic group, educational level, civil status, employment status and clustering of subjects within weeks of presence at Dallund Rehabilitation Centre.

The levels for both groups combined can be approximately computed from the table; considering total mood disturbance for example, the baseline level for both groups combined equals (16.8 × 433/794)+(17.5 × 361/794)=17.1; the follow-up 1 level equals ((16.8–4.9) × 433/794)+((17.5–3.6) × 361/794)=12.8; the difference between follow-up 1 and baseline equals 12.8–17.1=−4.3; and similarly for the other time points and quality of life indicators.

a Follow-up 1 at 1 month.

b Follow-up 2 at 6 months.

c Follow-up 3 at 12 months.

d Linear model includes additional adjustment on baseline score.

**Table 4:** Baseline level and evolution of mean quality of life indicators for 194 active participants and 167 non-active participants in the intervention group in the study of Internet support in cancer rehabilitation, Denmark, 2004–2006

	**Active in support group (*n*=194)**				**Not active in support group (*n*=167)**				**Significance level of tests**			
		**Difference between follow-up and baseline level**				**Difference between follow-up and baseline level**						
**Measure**	**Baseline level**	**1** a	**2** b	**3** c	**Baseline level**	**1** a	**2** b	**3** c	**Baseline**	**1** d	**2** d	**3** d
*Profile of mood states*												
Total mood disturbance	15.7	−4.0	−4.6	−6.8	19.7	−3.1	−3.0	−6.7	NS	NS	NS	NS
Anger/hostility	3.0	−0.6	−0.5	−0.7	3.0	−0.1	0.0	−0.4	NS	NS	NS	NS
Confusion/bewilderment	4.0	−0.2	0.1	−0.5	4.4	0.1	0.1	−0.5	NS	NS	NS	NS
Depression/dejection	5.7	−0.6	−0.9	−1.1	6.9	−0.9	−0.6	−1.8	NS	NS	NS	NS
Fatigue/inertia	7.3	−1.1	−1.2	−1.6	7.6	−0.6	−0.7	−0.8	NS	NS	NS	NS
Tension/anxiety	6.0	−0.6	−0.7	−1.5	6.8	−1.1	−1.0	−1.5	NS	NS	NS	NS
Vigour/activity	10.5	0.7	1.4	1.5	9.1	0.5	0.8	1.6	NS	NS	NS	NS
												
*Mental adjustment to cancer*												
Anxious preoccupation	20.1	−0.9	−1.1	−1.9	20.5	−1.2	−0.9	−1.7	NS	NS	NS	NS
Avoidance	9.5	−0.1	0.0	−0.1	10.3	−0.1	−0.1	0.0	NS^**^	NS	NS	NS
Fatalism	13.3	−0.2	−0.2	−0.2	13.5	0.0	0.0	−0.4	NS	NS	NS	NS
Fighting spirit	11.9	0.1	0.1	0.3	11.5	0.1	−0.1	−0.2	NS^*^	0.03	0.05	0.04
Helplessness	12.5	−0.3	−0.6	−0.9	13.9	−0.9	0.0	−0.9	NS^**^	NS	NS	NS
Self-rated health	3.2	−0.2	−0.2	−0.3	3.4	−0.1	−0.1	−0.2	0.04^**^	NS	NS	NS

Numbers are numbers of persons who completed the baseline questionnaire.

Significant in unadjusted *t*-tests at ^*^P<0.05, ^**^P<0.01, NS, not significant at *P*<0.05. Numbers and NS refer to the *P*-value in the F-test of the difference between groups in a linear regression model adjusted for sex, age, diagnostic group, educational level, civil status, employment status and clustering of subjects within weeks of presence at Dallund Rehabilitation Centre.

a Follow-up 1 at 1 month.

b Follow-up 2 at 6 months.

c Follow-up 3 at 12 months.

d Linear model includes additional adjustment on baseline score.
