# Supplementary material for: Effect of Internet peer-support groups on psychosocial adjustment to cancer: a randomised study
Source: Br J Cancer. 2010 Apr 27;102(9):1348–54. doi: 10.1038/sj.bjc.6605646 (PMC2865756; doi:10.1038/sj.bjc.6605646)
Supplement: Supplementary Table 1 [file 6605646x1.pdf]

Supplement 1. Randomized studies published 1990–2008 of the effect of psychosocial Internet-based support interventions on anxiety, depression, global quality of life and other findings in cancer patients

Study authors<br>Study location	Cancer site<br>No. of patients	Intervention or type of support group<br>Duration of intervention<br>Follow-up	Findings			
			Anxiety	Depression	Quality of life	Others
Gustafson *et al.* 2001 (4)<br>Wisconsin and Illinois, USA	Breast cancer, stage 1 or 2<br>246	CHESS'[a] program: information services (e.g. questions and answers, instant library); support services (discussion groups, bulletin board, ask the expert, personal stories); decision services (assessment, health charts, decision aid, action plan). Professional moderation<br>26 weeks<br>2 and 5 months	Not assessed	Not assessed	FACT-B; NS	Information competence: name of scale not indicated: $p = < 0.01$[b]<br>Interaction with health care system, level of comfort: name of scale not indicated: $p = < 0.01$[c]<br>Confidence in doctor: FACT-B (single item): $p = < 0.05$[c]<br>Social support: name of scale not indicated: $p = < 0.01$[d]
Winzelberg *et al.* 2003 (5)<br>California, USA	Breast cancer<br>72	'Bosom Buddies': Structured, web-based support group (discussion group, reading personal stories, web-based personal journal). Professional moderation<br>12 weeks<br>Once at 12 weeks	STAI; NS	CES-D; $p = 0.01$	Not assessed	Post-traumatic stress disorder: PCL-C: $p = < 0.01$<br>Global perceived stress: PSS: $p = < 0.05$<br>Cognitive–behavioral coping: CBI: NS<br>Adjustment to cancer: Mini-MAC: NS
Owen *et al.* 2005 (6)<br>California, USA	Breast cancer, stage 0 to 3<br>53	'SURVIVE' web site (bulletin board, dictionary of medical terminology, database of breast cancer resources, information and coping advice for physical symptoms, forum for sharing artwork and poetry, six structured coping skills training exercises). Self-guided<br>12 weeks<br>Once at 12 weeks	Not assessed	Not assessed	FACT-B; NS	Self-rated health: EuroQol-5D: $p = < 0.001$<br>Distress: IES: NS<br>Psychological well-being: FACT-EWB: NS<br>Physical well-being: MSAS: NS
Current study<br>Copenhagen, Denmark	12 different cancer sites<br>788	'Dallund' groups (26 groups, each including a discussion forum, live chat room, personal message system). Self-guided<br>12 months<br>1, 6 and 12 months	POMS tension-anxiety: NS	POMS depression–dejection: $p = 0.04$ at 6-month follow-up and increased in intervention group	Not assessed	Self-rated health: single item: NS<br>Adjustment to cancer: Mini-MAC, helplessness: $p = 0.002$ at 6-month follow-up; anxious preoccupation: $p = 0.04$ at 6-month follow-up' both increased in intervention group<br>POMS: confusion/bewilderment: $p = 0.001$ at 6-month follow-up and increased in intervention group; vigor/activity $p = 0.001$ at 12-month with higher increase in intervention group.

NS, not significant; FACT-B, Functional Assessment of Cancer Therapy–breast cancer; FACT-EWB, FACT–emotional well-being; STAI, Spielberger State/Trait Anxiety Inventory; CES-D, Center for Epidemiological Studies–depression; PCL-C, Posttraumatic Stress Disorder Checklist–civilian version; PSS, Perceived Stress Scale; CBI, Cancer Behavior Inventory; Mini-MAC, Mini Mental Adjustment to Cancer; EuroQol-5D, feeling thermometer; IES, Impact of Events Scale; MSAS, Memorial Symptom Assessment Scale; POMS, Profile of Mood States.

[a] At the time of study, the Comprehensive Health Enhancement Support System (CHESS) program was not yet Internet-based but functioned as a computer program on a home computer with a modem connection to a central server.

[b] Significant at 2-month follow-up; effect had decreased but not disappeared at 5-month follow-up

[c] Significant at 2-month follow-up; effect had disappeared at 5-month follow-up

[d] Significant at 5-month follow-up
