# Supplementary material for: Effect of Internet peer-support groups on psychosocial adjustment to cancer: a randomised study
Source: Br J Cancer. 2010 Apr 27;102(9):1348–54. doi: 10.1038/sj.bjc.6605646 (PMC2865756; doi:10.1038/sj.bjc.6605646)
Supplement: Supplementary Table 2 [file 6605646x2.pdf]

Supplement 2. Evolution of mean quality of life (QOL) measures between follow-ups and baseline in relation to total level of activity up to follow-up time of the active participants in the study of internet support in cancer rehabilitation, Denmark, 2004–2006

Measure	Change in QOL indicators,					
	Between follow-up at 1 month and baseline[1]		Between follow-up at 6 months and baseline[2]		Between follow-up at 12 months and baseline[3]	
	Coefficient (per 10 postings)	95% CI	Coefficient (per 10 postings)	95% CI	Coefficient (per 10 postings)	95% CI
Profile of mood states						
Total mood disturbance	0.54	(–0.39 ; 1,48)	0.21	(–0.19 ; 0.61)	0.55	(0.24 ; 0.87)
Anger/hostility	0.01	(–0.17 ; 0.19)	0.02	(–0.07 ; 0.10)	0.07	(–0.01 ; 0.14)
Confusion/bewilderment	0.00	(–0.11 ; 0.10)	–0.01	(–0.07 ; 0.06)	0.04	(–0.01 ; 0.09)
Depression/dejection	0.07	(–0.22 ; 0.37)	0.07	(–0.02 ; 0.16)	0.13	(0.06 ; 0.20)
Fatigue/inertia	0.04	(–0.09 ; 0.16)	0.02	(–0.06 ; 0.10)	0.13	(0.03 ; 0.23)
Tension/anxiety	0.18	(–0.03 ; 0.39)	0.09	(0.00 ; 0.17)	0.11	(0.07 ; 0.16)
Vigor/activity	0.28	(0.06 ; 0.49)	0.03	(–0.14 ; 0.21)	0.10	(–0.06 ; 0.25)
Mental adjustment to cancer						
Anxious preoccupation	0.05	(–0.15 ; 0.24)	0.01	(–0.10 ; 0.11)	–0.01	(–0.07 ; 0.05)
Avoidance	–0.11	(–0.22 ; 0.01)	–0.02	(–0.08 ; 0.04)	0.01	(–0.04 ; 0.06)
Fatalism	–0.02	(–0.10 ; 0.07)	0.03	(–0.01 ; 0.07)	0.02	(–0.05 ; 0.08)
Fighting spirit	–0.04	(–0.11 ; 0.03)	0.02	(–0.03 ; 0.06)	–0.03	(–0.07 ; 0.01)
Helplessness	–0.02	(–0.25 ; 0.21)	–0.01	(–0.10 ; 0.08)	0.05	(0.00 ; 0.10)
Self-rated health	0.04	(0.00 ; 0.07)	0.00	(–0.01 ; 0.02)	0.02	(0.00 ; 0.05)

CI, confidence interval
All models adjusted on baseline score, sex, age, diagnostic group, educational level, civil status, and employment status, and for clustering of subjects within weeks of presence at Dallund Rehabilitation Centre
[1] Analyses based on 188 active participants who completed both baseline and F1 questionnaire
[2] Analyses are based on 172 active participants who completed both baseline and F2 questionnaire
[3] Analyses based on 163 active participants who completed both baseline and F2 questionnaire
